# Management of suicidal crisis in an adolescent with Becker muscular dystrophy: a case report

**DOI:** 10.1186/s12888-026-08139-1

**Published:** 2026-04-30

**Authors:** Hongying Wan, Liyun Zheng, Xiaopeng Wu, Jianwen Xiong, Chaoxiong Zhou

**Affiliations:** 1 Department of Psychiatry, Jiangxi Mental Hospital & Affiliated Mental Hospital, Jiangxi Medical College, Nanchang University, Nanchang, Jiangxi 330029 China; 2 Nanchang City Key Laboratory of Biological Psychiatry, Jiangxi Provincial Clinical Research Center on Mental Disorders, Jiangxi Mental Hospital, Nanchang, Jiangxi 330029 China

**Keywords:** Becker muscular dystrophy, Suicidal ideation, Self-injurious behavior, Suicide attempt, Adolescent, Case report

## Abstract

**Background:**

Becker muscular dystrophy (BMD) is a rare X-linked recessive disorder associated with progressive physical disability, which may increase the risk of suicidal ideation. However, acute suicidal crises are rarely reported in BMD, as neuromuscular vulnerability restricts standard emergency pharmacological and somatic interventions. We describe an adolescent with BMD complicated by severe suicidal crisis to highlight key diagnostic and therapeutic considerations.

**Case presentation:**

A 16-year-old boy, who was diagnosed with BMD at the age of 8, became socially isolated due to progressive muscle weakness and long-term home confinement. He had experienced depressed mood for 3 years and recurrent self-injurious behavior for 6 months before admission, eventually engaging in wrist-cutting and an attempted jump from a building. Psychological assessment revealed severe depression and anxiety. To avoid premature definitive classification during an acute crisis, the patient was managed under a broader working diagnosis of mood disorder. Treatment emphasized psychotherapy and family intervention, with adjunctive low-dose fluoxetine (20 mg/day) and olanzapine (5 mg/day) to control impulsivity while minimizing sedation-related worsening of muscle weakness. Suicidal ideation resolved within 5 days, and no further severe suicidal behavior occurred over 10 months of follow-up.

**Conclusions:**

Severe suicidal behavior in BMD highlights the profound psychiatric impact of progressive physical and social decline. In adolescents with BMD and severe suicidal behaviors, dynamic assessment and early crisis intervention are essential. Psychological and family-based interventions should be prioritized, while pharmacotherapy, when needed, should be used cautiously to avoid exacerbating underlying neuromuscular weakness.

## Background

Becker muscular dystrophy (BMD) is a rare X-linked recessive disorder characterized by progressive muscle weakness, calf pseudohypertrophy, and impaired motor function [1, 2]. In addition to neuromuscular symptoms, patients with BMD may experience psychiatric comorbidities, including depression, anxiety, attention-deficit/hyperactivity disorder, and obsessive-compulsive disorder [3, 4]. A recent narrative review further highlights the significant psychological burden of neuromuscular diseases, encompassing high rates of anxiety, depression, maladaptive coping strategies, and diminished quality of life [5]. The psychological burden of physical disability, together with bullying, social exclusion, and functional decline, may further increase the risk of suicidal ideation [4, 6]. While mild-to-moderate psychological distress is well-recognized, progression to an acute suicidal crisis with habitual self-injury remains a severe and clinically underreported presentation [4, 6, 7].

Psychological assessment and intervention are important for improving quality of life in this population and may also contribute to suicide prevention [7, 8]. Recent work has introduced the Behavior, Emotion, Learning, and Social (BELS) screening tool for children with BMD, highlighting the value of early identification and intervention for psychological difficulties [8]. Rehabilitation approaches, such as moderate resistance training, may also help relieve depression and anxiety and improve overall well-being [9]. However, evidence remains scarce on the management of patients with BMD who present with acute suicidal crisis [6, 7].

Treating such emergencies is particularly challenging because many standard high-efficacy interventions for acute suicidality are substantially limited in this population [10–13]. The sedative and motor adverse effects of conventional psychotropic medications may further impair residual mobility and increase the risk of falls [10, 11]. In addition, physical treatments such as modified electroconvulsive therapy may carry unacceptably high anesthetic and neuromuscular risks in patients with muscular dystrophy [12, 13]. These constraints can significantly hinder timely stabilization.

To address this gap, we report the case of an adolescent with BMD who presented with intense suicidal ideation and suicidal behaviors. By describing his clinical presentation, psychological assessment, and treatment course, we aim to highlight key diagnostic considerations and intervention strategies that navigate this severe therapeutic dilemma while ensuring strict neuromuscular safety for this high-risk population.

## Case presentation

The patient was a 16-year-old boy with Becker muscular dystrophy (BMD) diagnosed in childhood. At age 8, he presented to a children’s hospital with lower-extremity weakness, reduced walking endurance, and leg pain after walking. High-throughput sequencing with chip capture identified a hemizygous deletion in the DMD gene (EX45_47DEL), a known pathogenic in-frame mutation consistent with the BMD phenotype [14]. Electromyography showed a myopathic pattern, and creatine kinase levels were markedly elevated. Physical examination revealed bilateral gastrocnemius pseudohypertrophy, preserved ability to rise from squatting, and a negative Gowers’ sign. Based on these findings, a neurologist confirmed the diagnosis of becker muscular dystrophy and issued a formal diagnostic certificate.

As the disease progressed, he gradually lost opportunities for school participation and peer interaction, became increasingly socially withdrawn, and struggled academically. He eventually left school voluntarily in the second year of middle school. Thereafter, he spent most of his time alone at home using electronic devices. Because his parents worked away from home for long periods, he was mainly cared for by his grandfather. Over the following 3 years, he developed persistent low mood, loss of interest, and marked social avoidance. Of note, there was no reported family history of medical or psychiatric illnesses. However, formal pedigree verification and maternal carrier testing were not performed; therefore, it remains unclear whether the pathogenic mutation was maternally inherited or arose *de novo*. Despite his progressively worsening mental state during this period, he had not received any prior psychiatric or psychological treatment.

Six months before admission, he began repeatedly hitting both temporal regions with his fists or hard objects, often leaving visible bruises. Although these episodes could be temporarily stopped by family members, they recurred once or twice weekly. Four days before admission, the behavior escalated. He struck his head forcefully with a smartphone and repeatedly hit his head against the wall, requiring several family members to physically restrain him. During an episode of emotional dysregulation, he also attempted wrist-cutting and tried to jump from a building, but both acts were interrupted by his family. He was then brought to the psychiatric emergency ward because of acute suicidal crisis.

On admission, psychiatric evaluation showed marked depression, helplessness, and self-blame. He reported longstanding social fear and feelings of inferiority, accompanied by recurrent and uncontrollable thoughts of self-harm and suicide. On structured clinical assessment of suicidality, the patient endorsed active suicidal ideation with high intensity and frequency. He described a desire to die and expressed that death would be a relief from his suffering, though he denied having formulated a specific plan or selected a method in advance. The recent suicide attempts (wrist-cutting and attempted jump) were characterized as impulsive acts during episodes of acute emotional dysregulation rather than premeditated behavior. He stated that these thoughts had recently intensified in both frequency and duration, culminating in the escalation from repetitive self-injury to suicide attempts. Further interview suggested that his social anxiety was rooted in a persistent sense of worthlessness, which made him fearful of leaving home or encountering strangers. Prolonged isolation appeared to aggravate his hopelessness about the future and intensify negative affect. He also described self-injury as a habitual means of emotional release, although it provided no lasting relief and was followed by increasingly frequent negative thoughts.

The patient obtained a standard score of 56 on the Zung Self-Rating Depression Scale (SDS) and a standard score of 69 on the Zung Self-Rating Anxiety Scale (SAS), which quantified the severity of his severe depressive and anxiety symptoms. Cognitive and personality assessments were also performed. On the Wechsler Intelligence Scale for Children, his Full-Scale IQ was 80, with a relatively higher Performance IQ of 86 and a borderline Verbal IQ of 76. The Minnesota Multiphasic Personality Inventory suggested introversion, loneliness, low self-esteem, and social anxiety. Physical examination showed lower-extremity muscle hypertrophy with reduced strength and a clumsy gait. Laboratory testing revealed markedly elevated creatine kinase levels and mild liver enzyme abnormalities. Echocardiography showed a left ventricular ejection fraction (LVEF) of 59% and a fractional shortening (FS) of 30%, within normal limits, though at the lower boundary, and the electrocardiogram (ECG) showed sinus arrhythmia.

Based on the clinical interview, psychometric assessment, and International Classification of Diseases, 10th Revision (ICD-10) diagnostic criteria, depressive disorder was identified as the primary diagnostic consideration, given the patient’s persistent low mood, anhedonia, social withdrawal over more than three years, and acute suicidal crisis. Subsequently, additional differential diagnoses were considered. Bipolar depression was evaluated but considered unlikely in the absence of any current or historical evidence of manic or hypomanic episodes. However, given the early age of onset, the possibility of a future manic switch could not be entirely excluded. Social anxiety disorder was considered in view of his marked fear of leaving home, feelings of inferiority, and avoidance of strangers. Adjustment disorder was likewise considered, given the apparent psychological response to progressive physical decline and school dropout. Obsessive-compulsive disorder (OCD) was also considered, as the patient’s recurrent, uncontrollable self-injurious behavior could overlap with obsessive-compulsive spectrum phenomena. However, the self-injury appeared primarily mood-driven and lacked ritualistic features or ego-dystonic obsessions characteristic of OCD. Attention-deficit/hyperactivity disorder was considered as a potential contributor to academic failure and social impairment [15], though no prominent symptoms of inattention or hyperactivity/impulsivity independent of the depressive presentation were observed.

However, distinguishing clearly among these diagnoses was clinically difficult. His social avoidance appeared to be closely related to the stigma and functional limitations associated with BMD rather than to a primary anxiety disorder. In addition, differentiating severe maladjustment from major depression in the context of a chronic progressive neuromuscular disease was inherently complex. Because imposing a definitive diagnostic label during an acute suicidal crisis risked both premature classification and additional stigma, a broader working diagnosis of mood disorder was adopted. This approach prioritized immediate safety, symptom stabilization, and targeted psychosocial intervention, while allowing for longitudinal observation to clarify the definitive diagnosis.

Treatment began with 24-hour safety precautions and close observation. A nurse trained in psychological counseling provided daily 1-hour supportive sessions. On the day of admission, fluoxetine 20 mg/day was initiated for depressive symptoms, together with olanzapine 5 mg/day to reduce impulsivity and the immediate risk of self-harm and suicide. Given his severe social avoidance and reliance on self-injury as a maladaptive coping strategy, structured psychological intervention was started concurrently. A cognitive behavioral therapy (CBT) framework was used to target negative automatic thoughts and social avoidance, and family intervention was introduced to help his family members recognize warning signs and establish a crisis response plan.

After a 5-day inpatient stay, his mood became more stable, suicidal ideation was markedly reduced, and sleep improved. At discharge, the diagnoses were Becker muscular dystrophy and mood disorder. During outpatient follow-up, CBT was continued at 2 weeks, 4 weeks, and then monthly for 10 months. Throughout this 10-month period, the patient demonstrated good treatment adherence. The initial medication regimen was maintained without dose adjustments. During the inpatient stay, adverse effects were systematically monitored using the Treatment Emergent Symptom Scale (TESS), and no extrapyramidal symptoms, abnormal involuntary movements, or akathisia were identified. Regarding tolerability, the patient reported mild drowsiness during the early phase of treatment, which was well tolerated and subsided without further intervention. During outpatient follow-up, clinical assessment for extrapyramidal symptoms and other significant adverse effects was conducted at each visit, with no abnormalities detected. The patient’s family reported an approximate weight increase of 2–3 kg over the 10-month follow-up period. Given his age (16–17 years), this modest change could not be definitively attributed to olanzapine versus normal adolescent growth. ECGs were repeated at follow-up visits, showing normal sinus rhythm, sinus arrhythmia, or sinus bradycardia, findings generally considered benign in this age group. Echocardiographic monitoring was not repeated during this period.

**Fig. 1:**
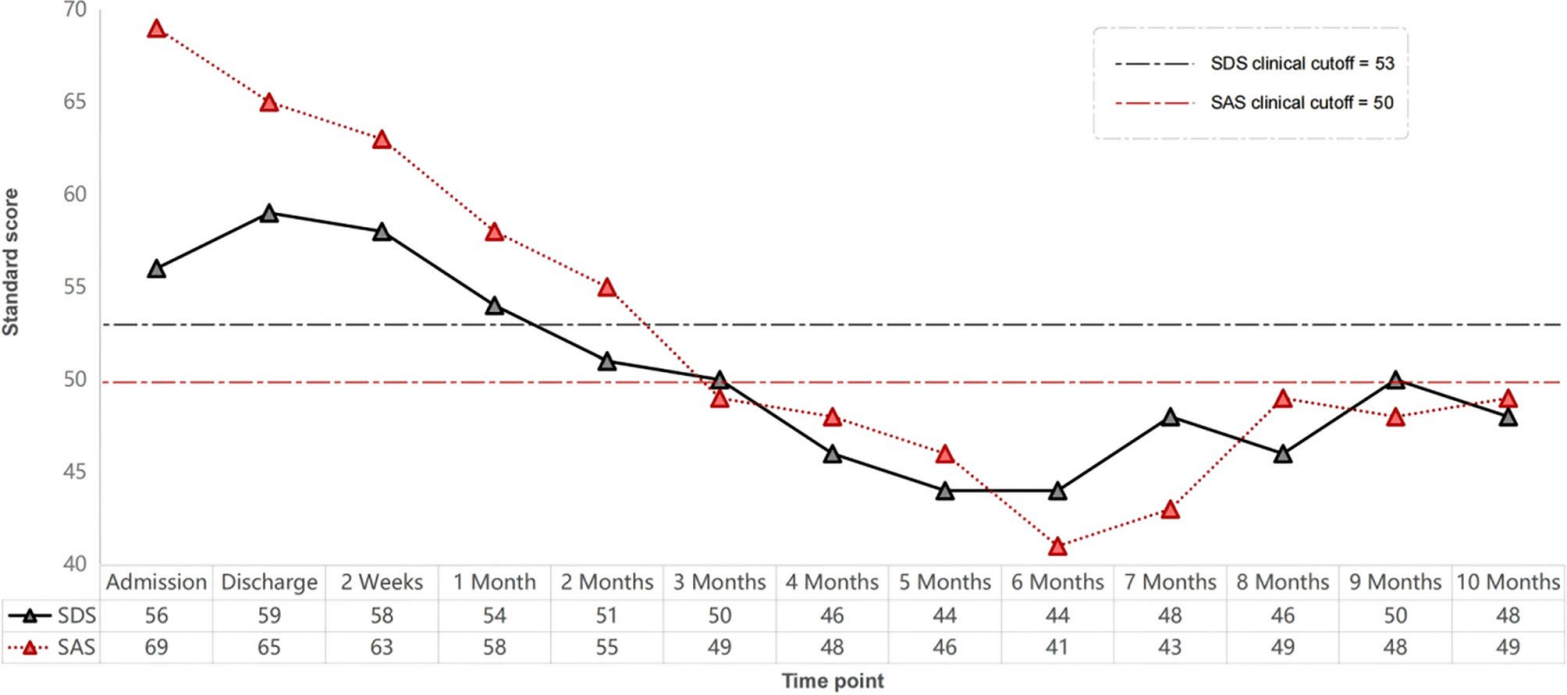
Line graph of Zung Self-Rating Depression Scale (SDS) and Zung Self-Rating Anxiety Scale (SAS) scores from admission through 10-month follow-up

The SDS and SAS were administered at each follow-up visit. During the first month post-discharge, both scores remained above the established clinical cutoffs (SDS standard score ≥ 53; SAS standard score ≥ 50) [16]. The SDS score fell below the clinical threshold by the second month of follow-up, while the SAS score normalized by the third month. Both scores subsequently remained within the normal range, though consistently near the upper boundary, likely reflecting the persistent influence of biopsychosocial factors associated with BMD (Fig. 1). Regarding suicidality, structured reassessment at each visit confirmed progressive resolution of active suicidal ideation. The patient reported only occasional fleeting thoughts of self-harm without intent or plan, and no further severe suicidal behavior occurred during the 10-month follow-up (Table 1).

**Table 1:** Timeline of Clinical Events and Interventions

Timepoint	Clinical Event	Management / Intervention
Age 8	Diagnosed with Becker muscular dystrophy (BMD) via genetic testing after presenting with muscle weakness.	Routine physical follow-up; no psychiatric intervention.
Age ~ 13	Voluntarily left school due to physical progression and academic struggles; began severe social withdrawal.	Home care primarily by grandfather; prolonged use of electronic devices.
Age 13–16	Developed progressive low mood, loss of interest, and marked social avoidance.	No psychiatric or psychological treatment received.
6 Months Prior to Admission	Onset of self-injurious behavior (hitting head with fists/hard objects 1–2 times/week).	Family members attempted verbal persuasion and temporary physical interruption.
4 Days Prior to Admission	Escalation of self-injury (hitting head against walls/with smartphone); attempted wrist-cutting and jumping from a building.	Family physically restrained the patient and brought him to the psychiatric emergency ward.
Admission	Severe depressive and anxiety symptoms, feelings of worthlessness, acute suicidal crisis.	24-hour safety precautions; Fluoxetine 20 mg/d & Olanzapine 5 mg/d; daily 1-hour supportive sessions; CBT framework initiated. Adverse effects monitored using TESS (no extrapyramidal symptoms detected). Echocardiography: LVEF 59%, FS 30%; ECG: sinus arrhythmia.
Day 5 (Discharge)	Mood stabilized, suicidal ideation markedly reduced, sleep improved. Diagnosed with BMD and mood disorder.	Discharged with outpatient follow-up plan and psychotropic medication maintenance.
10-Month Follow-up	SDS and SAS scores normalized by months 2 and 3, respectively, remaining near upper boundary. Only occasional fleeting thoughts of self-harm; no severe suicidal behavior occurred; no severe suicidal behavior occurred. Approximate weight gain of 2–3 kg (confounded by normal adolescent growth).	1-hour CBT-based psychotherapy & clinical follow-up at weeks 2 and 4, then monthly, with structured suicidality reassessment and clinical monitoring for extrapyramidal symptoms at each visit. Follow-up ECGs: normal sinus rhythm/sinus arrhythmia/sinus bradycardia.

## Discussion

While the typical clinical presentation of BMD occurs during late adolescence or early adulthood, studies in Chinese cohorts suggest an earlier age of diagnosis, with the majority of patients identified before the age of 11 [17]. In this 16-year case, progressive muscle weakness and motor impairment caused by BMD gradually limited the patient’s participation in school and peer activities, leading to increasing social isolation and heightened vulnerability to depression and anxiety [5]. The cumulative impact of these biopsychosocial factors ultimately contributed to the emergence of severe suicidal behavior. This course is consistent with previous findings that patients with BMD are at increased risk of depression, anxiety, and cognitive difficulties [3, 4, 18, 19].

Although the patient’s symptoms strongly aligned with the diagnostic criteria for depressive disorder, several considerations warranted caution before assigning a definitive classification. The patient is an adolescent navigating a developmental stage characterized by heightened emotional sensitivity regarding self-identity, body image, and peer relationships, which may color the clinical presentation [20]. Moreover, the persistent psychosocial impact of BMD, coupled with developing emotional regulation capacities, may constitute the primary driver of depressive symptoms rather than a primary depressive disorder independent of the psychosocial context [21, 22]. The possibility of an emerging bipolar disorder in early-onset depression further necessitates longitudinal observation before definitive classification [23]. In this case, the broad working diagnosis of mood disorder provided a practical basis for emergency safety measures, mood stabilization, and antidepressant treatment, reflecting diagnostic prudence in a genuinely complex clinical scenario rather than an avoidance of diagnostic clarity.

From a suicidological perspective, the trajectory from chronic distress to acute suicidal crisis in this patient can be understood through established theoretical frameworks [24]. The patient’s suicidal impulses were triggered when intense negative emotions could not be effectively regulated, a process driven by the interplay of multiple factors [25]. Chronic situational stress, arising from prolonged exposure to the functional limitations and social exclusion associated with BMD, generated accumulating negative affect [5, 7]. The patient’s negative self-appraisal and pervasive hopelessness regarding the future constituted additional sources of persistent emotional distress [26]. Crucially, his developing emotional regulation capacities were insufficient to tolerate or express these accumulating negative emotions, ultimately giving rise to an overwhelming impulse to end his suffering [27].

Self-injury initially served as the patient’s maladaptive coping strategy for managing negative emotions, consistent with Nock’s functional model of non-suicidal self-injury, in which self-harm operates as a means of automatic negative reinforcement, providing temporary relief from overwhelming distress [28]. However, this relief was transient. The self-injurious behavior failed to produce lasting benefit and was followed by increasingly frequent negative thoughts [29]. As the patient progressed from suicidal ideation to repetitive self-harm, his tolerance for pain, fear, and the concept of death gradually increased. This trajectory is consistent with Joiner’s Interpersonal-Psychological Theory of Suicide, which posits that repeated exposure to painful and provocative experiences facilitates the development of acquired capability for suicide, through progressive habituation to pain and diminished fear of death, thereby enabling the escalation from ideation to action [30, 31].

Pharmacotherapy in BMD requires additional caution because of the underlying neuromuscular vulnerability. Indeed, any medication that carries the potential to exacerbate muscle weakness, compromise respiratory function, or induce excessive sedation requires heightened vigilance in patients with BMD, given the progressive muscle weakness and functional decline inherent to BMD [32]. Regarding drug selection, fluoxetine was chosen as the first-line antidepressant given its established evidence base for adolescent depression [33]. Low-dose olanzapine was added for its acute sedative and anxiolytic properties, its capacity to improve sleep, and its potential to reduce impulsivity [34]. In the maintenance phase, the fluoxetine-olanzapine combination was continued to improve mood state and as a precautionary measure given the need for longitudinal monitoring of possible mood switching in early-onset depression [35, 36].

However, olanzapine carries metabolic risks, including weight gain and glucose and lipid abnormalities, that may impose additional burden on patients with BMD, necessitating ongoing metabolic surveillance [37]. If significant metabolic adverse effects emerge, alternative strategies should be considered [36]. Lithium carbonate, with its established mood-stabilizing and anti-suicidal properties, represents one option, though it requires several weeks to take effect [38]. Besides, clinicians must remain vigilant regarding potential neuromuscular risks, as lithium can induce or exacerbate myasthenia-like symptoms [39]. Alternative augmentation agents with lower metabolic risk profiles, such as lurasidone, cariprazine, and aripiprazole, may also be considered [40, 41]. However, their relative lack of sedative properties may limit their utility in the acute phase, and the risk of extrapyramidal side effects warrants caution in patients with pre-existing motor impairment [42, 43].

Management of adolescents with BMD and severe suicidal crisis should extend beyond a single treatment modality. A combined intervention strategy is needed, with psychotherapy as the foundation and pharmacotherapy as an adjunct [33, 44, 45]. In this patient, the core distress was closely linked to social isolation, stigma, and loss of function related to his physical illness. Medication alone would have been insufficient to address these psychosocial drivers of suffering. CBT was introduced to address feelings of worthlessness, negative automatic thoughts, and social avoidance, while family intervention focused on improving supervision, recognizing warning signs, and establishing a crisis response plan [45, 46]. Beyond individual therapy, group-based interventions may further foster social confidence and reduce isolation, though such approaches may be premature during the acute phase and would require gradual introduction as symptoms stabilize [47, 48]. Adjunctive family support groups, particularly for caregivers of individuals with rare neuromuscular diseases, may also help address the supervision and crisis-response challenges illustrated in this case [49].

The resolution of suicidal ideation within five days warrants cautious interpretation with regard to causal attribution. Given the patient’s maladaptive coping patterns and developing emotional regulation capacities, the intensive psychological support and cognitive restructuring provided during the inpatient stay likely played a primary role. The sedative and sleep-improving properties of low-dose olanzapine may have provided supportive early symptom relief. Fluoxetine, given its well-established delayed onset of antidepressant action, is unlikely to have contributed meaningfully within this timeframe [50]. Additional contributing factors include the containing effect of the structured inpatient environment and the natural emotional stabilization that commonly follows an acute suicidal crisis. Notably, while acute suicidal crisis resolved within five days, the SDS and SAS scores remained above clinical cutoffs at discharge, supporting the interpretation that the early improvement reflected crisis containment and emotional stabilization rather than treatment of the mood disorder itself.

## Strength and Limitation

The main strength of this case report is the longitudinal observation of a rare and complex psychiatric presentation in an adolescent with BMD, together with the successful use of a multimodal treatment strategy. By combining cautious low-dose pharmacotherapy with targeted CBT and family intervention, we addressed not only the acute suicidal crisis but also the underlying psychosocial factors contributing to his distress.

Several limitations should also be noted. First, as a single case report, the findings are inherently limited in generalizability and lack a controlled comparison. Second, although the patient showed symptomatic improvement during 10 months of follow-up, this period remains relatively short for assessing the long-term course of a chronic progressive neuromuscular disorder. Third, the patient’s psychosocial context, being primarily cared for by his grandfather while his parents worked away from home, reflects a specific family structure that may not be generalizable across different cultural or healthcare settings. Fourth, group-based psychotherapy and adjunctive caregiver support groups were not available in our clinical setting; such resources may offer additional benefits for social rehabilitation and family support in similar cases.

Additionally, the assessment battery was constrained by institutional and resource limitations, resulting in exclusive reliance on self-report symptom measures without complementary clinician-administered instruments such as the Hamilton Depression Rating Scale or Hamilton Anxiety Rating Scale. Similarly, standardized tools for evaluating specific domains, including suicidality (e.g., Columbia Suicide Severity Rating Scale), obsessive-compulsive symptoms (e.g., Yale-Brown Obsessive Compulsive Scale), attention-deficit/hyperactivity disorder (e.g., Conners scale, SNAP-IV), and antipsychotic-related movement disorders (e.g., Abnormal Involuntary Movement Scale, Barnes Akathisia Scale), were not employed. This reliance on clinical interviews and general rating scales, while consistent with routine practice in our setting, may have limited the precision of diagnostic differentiation and the sensitivity of adverse effect detection. Comprehensive use of such standardized instruments is recommended in future assessments of similar cases.

Furthermore, systematic physiological monitoring was incomplete during the 10-month follow-up. Weight and metabolic parameters were not formally tracked, despite the well-established metabolic risks associated with long-term olanzapine use [37]. Echocardiographic surveillance was likewise not repeated during the psychiatric follow-up period. Given that BMD itself carries risks of cardiomyopathy and that olanzapine may compound metabolic burden, the absence of structured physiological monitoring represents a notable gap. Ongoing periodic metabolic and cardiac surveillance remains essential in this population, consistent with current management guidelines [1].

## Conclusions

This case highlights the importance of early recognition and comprehensive crisis intervention in adolescents with becker muscular dystrophy who present with severe psychiatric symptoms and suicidal behavior. Clinical assessment should emphasize diagnostic caution, dynamic observation, and symptom-focused management rather than premature diagnostic labeling. Treatment should prioritize psychological and family-based interventions, with pharmacotherapy used cautiously as an adjunct. When prescribing psychotropic medication, clinicians must remain alert to the neuromuscular vulnerability associated with BMD. Such an approach may improve psychiatric outcomes while preserving physical safety.

## Patient Perspective

During the outpatient follow-up, the patient shared his reflections on the treatment and his recovery journey. He noted that the psychological interventions helped him realize he was not “worthless,” teaching him to focus on his strengths rather than his physical limitations. He rediscovered his passion for reading and writing fiction, an area where he had some prior experience. He has consistently maintained this practice throughout the follow-up period, reporting that writing his thoughts down provides significant emotional relief and comfort. Looking forward, he expressed a renewed sense of purpose, hoping to further develop his writing skills and ultimately share his stories with a wider audience.

## Data Availability

The data supporting the findings of this case report are not publicly available due to the risk of compromising individual privacy. No publicly archived datasets were generated for this study.

## Abbreviations

BMD: Becker Muscular Dystrophy
SDS: Zung Self-Rating Depression Scale
SAS: Zung Self-Rating Anxiety Scale
CBT: Cognitive Behavioral Therapy
BELS: Behavior, Emotion, Learning, and Social
ICD−10: International Classification of Diseases, 10th Revision
ECG: Electrocardiogram
LVEF: Left Ventricular Ejection Fraction
FS: Fractional Shortening
TESS: Treatment Emergent Symptom Scale
OCD: Obsessive-compulsive Disorder
